# The diagnostic and prognostic value of auditory statistical learning ability in patients with disorders of consciousness

**DOI:** 10.3389/fneur.2026.1841501

**Published:** 2026-07-22

**Authors:** Mingyan Yu, Xiangyue Xiao, Junhua Ding, Mengying Zhao, Sara Cruz, Nantu Hu, Mingwei Zhang, Steven Laureys, Haibo Di, Yan Chen

**Affiliations:** 1 International Unresponsive Wakefulness Syndrome and Consciousness Science Institute, School of Basic Medicine, Hangzhou Normal University, Hangzhou, China; 2 Zhejiang-Belgium Joint Laboratory for Disorders of Consciousness, Hangzhou, China; 3 State Key Laboratory of Cognitive Science and Mental Health, Institute of Psychology, Chinese Academy of Sciences, Beijing, China; 4 Department of Education and Psychology, William James Center for Research, University of Aveiro, Aveiro, Portugal; 5 Coma Science Group, GIGA Consciousness and Centre du Cerveau, University Hospital of Liège, Liège, Belgium

**Keywords:** diagnosis, disorders of consciousness, electroencephalography, prognosis, statistical learning

## Abstract

**Introduction:**

Accurate assessment of residual cognition in patients with disorders of consciousness (DoC) remains challenging due to their limited behavioral responsiveness. Statistical learning (SL), defined as the ability to extract regularities from sensory input, reflects neural processing associated with residual cognition. This study examined how SL is preserved across different levels of consciousness and whether neural markers of SL can inform diagnosis and prognosis in patients with DoC.

**Methods:**

We employed an auditory paradigm with structured trisyllabic pseudowords and recorded electroencephalography (EEG) from 46 patients and 25 healthy controls. Neural entrainment to statistical regularities was quantified using frequency-tagging analysis.

**Results:**

The within-group effect pattern was observed in the healthy control group and in the minimally conscious state (MCS) group, whereas the unresponsive wakefulness syndrome (UWS) group did not show a significant effect. Moreover, stronger neural learning predicted better behavioral scores in patients with MCS, but not in patients with UWS. Further analyses showed that SL-related effect correlated significantly with behavioral scores and 6-month outcomes in the traumatic group, whereas no such association was observed in the non-traumatic group. Importantly, SL-based EEG models effectively distinguished healthy controls from patients and further differentiated MCS from UWS patients. Combining the SL model with clinical behavioral performance could enable a more precise assessment of patients’ level of consciousness.

**Discussion:**

These findings suggest that SL may require a minimal level of consciousness in the integration of structured regularities and that it may serve as an index for clinical diagnosis and prognosis in patients with DoC.

## 1 Introduction

Disorders of consciousness (DoC) refer to a state in which a person’s awareness of themselves and their environment is significantly diminished or absent following a severe brain injury (1, 2). The etiology of impaired consciousness is typically categorized as either traumatic brain injury (TBI) or non-traumatic brain injury (NTBI) (1). TBI results from external mechanical forces, whereas NTBI is generally related to medical conditions such as intracranial hemorrhage, cardiac arrest, or ischemic or hemorrhagic stroke (3). According to the degree of impairment, patients with prolonged DoC are classified as either unresponsive wakefulness syndrome (UWS) or minimally conscious state (MCS) (4, 5). Patients with UWS exhibit sleep–wake cycles without conscious awareness, whereas patients with MCS demonstrate reproducible signs of consciousness, such as following commands or exhibiting purposeful responses (6, 7). Accurate diagnosis and reliable prognostic predictions for these patients remain a persistent challenge in contemporary clinical neuroscience and rehabilitation medicine. Traditional behavioral assessment scales, such as the Coma Recovery Scale-Revised (CRS-R) (8), have inherent limitations when it comes to detecting cognitive functions in patients, particularly those with sensory or motor impairments (9, 10). To address these limitations, recent studies have increasingly used brain imaging or electrophysiological techniques to detect residual brain activity in patients with DoC (11–14). Among these techniques, electroencephalography (EEG) has gradually become the focus of research due to its portability, non-invasiveness, and feasibility in routine clinical settings (11, 15–18). There are typically three types of EEG-based experimental paradigms used to assess preserved consciousness in patients with DoC: active paradigms involving task execution; passive paradigms based on stimulus-evoked brain responses; and resting-state assessments that infer the conscious state from spontaneous neural activity (19). Unlike active tasks, which require sustained attention or the ability to follow instructions, passive paradigms allow neural processing to be assessed without demanding high-level cognitive engagement or active participation. Moreover, passive paradigms provide more direct, stimulus-related evidence of preserved cognitive processing than resting-state assessments do (20). Given the varying levels of brain injury among patients with DoC, passive paradigms appear to be a promising approach for assessing residual cognitive function.

Statistical learning (SL), which is commonly assessed in a passive paradigm, shows unique clinical potential. SL refers to the ability to extract patterns and structures from sensory input without explicit instruction or feedback (21, 22). This ability was first demonstrated in studies of infants using auditory speech sequences containing repeated trisyllabic pseudowords (23), which showed that infants could segment words by tracking transitional probabilities (TP) between syllables, thereby learning the underlying patterns. TP quantifies the likelihood that a given element (e.g., a syllable or tone) follows another specific element within a sequence (24). Over the following decades, researchers have demonstrated that SL occurs across multiple sensory modalities, such as vision (25) and touch (26), and across the lifespan, from infancy and childhood to adulthood (27). It has also been observed in various populations such as healthy individuals and those with post-stroke aphasia (28), confirming its role as a universal, cross-domain cognitive mechanism. Crucially, SL does not require overt guidance or high cognitive effort. It can occur through passive exposure to structured input, without demanding active participation or explicit instructions (29). In contrast, explicit learning demands active participation, sustained attention, and the ability to follow commands (30). These capacities are often severely impaired in patients with DoC. This makes SL technically well-suited for investigating residual cognitive processing in patients with DoC. Recent work has highlighted the potential of SL paradigms in revealing residual cognitive abilities in patients with DoC. For example, Xu et al. (31) used a frequency-tagging EEG approach involving real and reversed disyllabic word sequences, revealing that patients with MCS and patients with emergence from MCS exhibited neural tracking of transitional probabilities between syllables. Similarly, Benjamin et al. (32) showed that some patients with MCS could learn statistical regularities in trisyllabic pseudowords when presented with structured versus random auditory streams, whereas patients with UWS showed only a weak trend. Together, these findings highlight the potential of SL paradigms for probing residual cognitive processing in patients with DoC. However, the extent to which SL-related neural measures can be used as reliable quantitative tools for clinical diagnosis and prognosis in patients with DoC remains to be fully established. This uncertainty partly reflects ongoing debates regarding whether SL necessarily depends on conscious awareness. Some researchers argue that SL is an automatic process (33, 34). However, emerging evidence suggests that top-down attention mechanisms may play a crucial role in SL (35, 36). This issue is highly relevant to patients with DoC, as their levels of consciousness vary. This spectrum, ranging from unconsciousness (UWS) to minimal consciousness (MCS), enables us to investigate the effect of consciousness on SL. In this context, examining SL capacities across individuals with varying levels of consciousness offers a unique opportunity to clarify this theoretical debate.

To address these issues, the present study employed a passive EEG paradigm with trisyllabic pseudowords to examine the diagnostic and prognostic relevance of SL-related neural measures in patients with DoC. Neural responses were recorded during continuous exposure to structured auditory input, and frequency-domain markers were extracted to reflect the encoding of statistical regularities. Correlation analyses between EEG-based markers of SL and behavioral assessments across degrees of consciousness impairment and etiology of brain injury were performed to evaluate their relevance to clinical status and disease characteristics, as well as in relation to 6-month clinical outcomes. Predictive modelling was further conducted using a generalized linear model to assess the clinical utility of the neural markers. Building on these analyses, we sought to investigate whether patients with varying etiologies and levels of consciousness retain SL abilities and to assess how these neural signatures could contribute to quantitative approaches for diagnosis and prognostic evaluation.

## 2 Materials and methods

### 2.1 Participants

The present study involved patients with DoC and healthy controls (HC). All participants were right-handed native Mandarin speakers with hearing considered clinically intact. The study protocol was approved by the Ethics Committee of Hangzhou Normal University (approval No. 2022–1,109). Local institutional authorization was also obtained from each hospital prior to participant enrollment. Written informed consent was obtained from all healthy participants and patients’ caregivers before the experiment. The dataset is identical to that used in our prior publication (37).

#### 2.1.1 Patients

A total of 51 patients with DoC were recruited from three institutions: Shanghai Yongci Rehabilitation Hospital, rehabilitation units at Hangzhou Wujing Hospital, and Zhejiang Rehabilitation Medical Centre. The inclusion criteria for this study were as follows: (i) diagnosis of MCS or UWS according to the CRS-R; (ii) age over 18 years; (iii) more than 28 days since the brain injury occurred; (iv) presence of the auditory startle reflex; (v) no history of hearing or visual impairments prior to the brain injury occurring; (vi) no history of psychiatric or neurological disorders prior to the brain injury occurring; and (vii) no significant cranial bone defects. Following the exclusion of five patients due to poor EEG data quality, the final analysis included 46 patients. This cohort consisted of 25 patients with MCS (19 males, mean age 61.24 ± 12.41 years) and 21 patients with UWS (18 males, mean age 52.14 ± 15.64 years). In terms of injury etiology, the cohort included 16 patients with TBI and 30 patients with NTBI. The demographic and clinical characteristics of all patients are summarized in Table 1.

**Table 1:** Demographic and clinical information of included patients (n = 46).

Patient ID	Diagnosis	Age (years)	Gender	Etiology	Time since injury (months)	CRS-R at EEG recording	Diagnosis at 6 months	CRS-R at 6 months
MCS01	MCS-	29	M	TBI	10	10	Died	N/A
MCS02	MCS-	44	M	NTBI	35	11	N/A	N/A
MCS03	MCS-	66	M	TBI	11	12	N/A	N/A
MCS04	MCS-	67	M	TBI	14	10	MCS-	11
MCS05	MCS-	81	M	NTBI	16	11	N/A	N/A
MCS06	MCS-	59	F	NTBI	4	10	MCS-	10
MCS07	MCS+	70	F	TBI	14	19	Died	N/A
MCS08	MCS-	50	M	NTBI	18	9	MCS-	11
MCS09	MCS+	66	M	TBI	13	11	MCS-	10
MCS10	MCS+	66	F	TBI	15	18	EMCS	20
MCS11	MCS-	53	M	NTBI	8	12	MCS-	13
MCS12	MCS-	67	F	NTBI	12	12	MCS-	10
MCS13	MCS-	77	M	NTBI	20	12	UWS	7
MCS14	MCS+	72	F	NTBI	10	15	MCS+	15
MCS15	MCS-	51	M	TBI	12	11	MCS-	14
MCS16	MCS-	75	M	TBI	18	14	MCS-	10
MCS17	MCS-	50	M	NTBI	23	11	Died	N/A
MCS18	MCS-	51	M	NTBI	11	13	MCS-	10
MCS19	MCS-	68	M	NTBI	20	9	UWS	6
MCS20	MCS-	72	M	NTBI	19	12	MCS-	9
MCS21	MCS-	52	M	TBI	9	13	MCS-	13
MCS22	MCS-	66	M	NTBI	16	13	MCS-	12
MCS23	MCS-	64	F	NTBI	18	11	MCS-	9
MCS24	MCS-	71	M	NTBI	22	12	UWS	7
MCS25	MCS-	44	M	NTBI	10	9	MCS-	10
UWS01	UWS	75	M	NTBI	13	7	N/A	N/A
UWS02	UWS	71	M	TBI	10	5	Died	N/A
UWS03	UWS	37	M	NTBI	11	7	N/A	N/A
UWS04	UWS	34	M	NTBI	12	5	UWS	8
UWS05	UWS	42	M	NTBI	7	7	UWS	8
UWS06	UWS	41	M	NTBI	5	7	UWS	7
UWS07	UWS	33	M	TBI	15	7	UWS	7
UWS08	UWS	55	M	TBI	12	7	MCS-	9
UWS09	UWS	54	M	TBI	3	7	UWS	7
UWS10	UWS	73	M	NTBI	18	2	UWS	5
UWS11	UWS	25	F	NTBI	9	7	UWS	8
UWS12	UWS	52	M	TBI	15	7	N/A	N/A
UWS13	UWS	29	F	NTBI	10	7	UWS	7
UWS14	UWS	71	M	NTBI	32	8	UWS	6
UWS15	UWS	67	M	TBI	26	7	UWS	8
UWS16	UWS	55	M	NTBI	13	7	N/A	N/A
UWS17	UWS	56	M	NTBI	10	6	UWS	7
UWS18	UWS	68	M	TBI	22	7	UWS	6
UWS19	UWS	65	M	NTBI	10	7	UWS	5
UWS20	UWS	51	F	NTBI	16	7	UWS	8
UWS21	UWS	41	M	NTBI	21	7	UWS	6

DoC, disorders of consciousness; CRS-R, Coma Recovery Scale-Revised; UWS, unresponsive wakefulness syndrome; MCS, minimally conscious state without (MCS-) or with (MCS+) evidence of language function; F, female; M, male; NTBI, non-traumatic brain injury; TBI, traumatic brain injury; N/A, data not available.

#### 2.1.2 Healthy controls

A total of 26 HC participants were recruited from the local community. All had normal hearing and vision, and no history of psychiatric or neurological disease. One participant was excluded due to excessive body movements during the experiment. The final control group included 25 HC (16 males; mean age 58.60 ± 11.08 years). Shapiro–Wilk tests confirmed that age was normally distributed in each group (*p* > 0.05). Statistical analysis revealed no significant differences in age or gender between the control group and the two patient cohorts (age: one-way ANOVA, *F* = 2.893, *p* = 0.062; gender: chi-square test, *χ^2^* = 2.881, *p* = 0.237).

### 2.2 Clinical assessments

The CRS-R scores (8, 38) were used to assess the level of consciousness of the patients across six behavioral dimensions: auditory, visual, motor, oromotor/verbal, communication, and arousal. Each patient underwent at least five CRS-R assessments over a 10-day period. The highest score was used as the final score and included in the analysis.

### 2.3 Stimuli and procedures

We created five trisyllabic pseudowords that were made up of syllables from five common, meaningful real words (i.e., “cháng jǐng lù” for giraffe, “xī hóng shì” for tomato, “tuī xiāo yuán” for salesman, “diàn yùn dǒu” for iron, and “fǔ wò chēng” for push-up). The real words differed significantly in their initial and final phonemes and tones. By randomly rearranging the syllables of these real words, we created five new trisyllabic pseudowords (i.e., “cháng xiāo shì,” “xī wò dǒu,” “tuī jǐng chēng,” “diàn hóng lù,” “fǔ yùn yuán”). Each syllable was used in only one pseudoword and did not repeat. These new words have no real meaning and are not semantically linked to each other.

**Figure 1:**
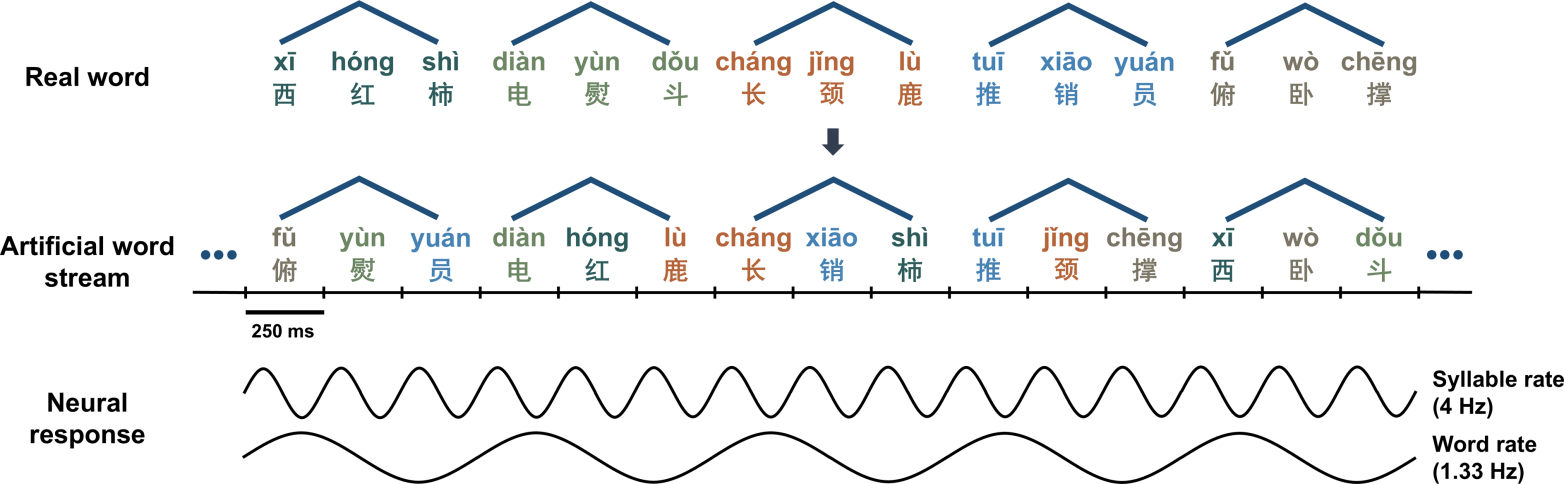
The pseudowords learning paradigm. The auditory stimulus stream contained five trisyllabic pseudowords. The pseudowords were created by randomly rearranging the syllables of five real Mandarin words. Each syllable lasted 250 ms, producing a constant rate of 4 Hz, while the pseudowords were presented at 1.33 Hz. Before exposure, word boundaries were not marked.

We then created a continuous speech by concatenating these pseudowords together in random order, ensuring there were no acoustic pauses between syllables and that identical words were not repeated immediately. Therefore, the TP between neighboring syllables is always 1 within each word and 0.25 at word boundaries. For example, a TP of 1.0 for the word “cháng xiāo shì” means that cháng always leads to xiāo and xiāo always to shì, while shì is equally likely to be followed by xī, tuī, diàn, and fǔ. The speech sequences contained a total of 2,400 syllables, corresponding to 800 artificial words, and were synthesized in a male voice using a Neospeech synthesizer.^1^ Importantly, the syllables were isochronous, each lasting 250 milliseconds. This resulted in a syllable rate of 4 Hz and a word rate of 1.33 Hz (Figure 1). Additionally, 7–8 random syllables were added to the beginning and end of the speech sequences. This design aimed to prevent participants from segmenting every three syllables as a word from the onset of the sequence while listening to the stimuli. The additional syllables added to the beginning and end of the speech sequences were randomly selected from those comprising the artificial words. The total duration of the speech sequences was thus 10 min and 3.75 s.

All stimuli were presented using the Psychtoolbox MATLAB toolbox (39). Participants listened to the speech sequences containing the pseudowords via headphones while their EEG responses were recorded. Participants were asked to listen attentively to the speech sequences without being given any information about the underlying structure of the stimuli and without undergoing any training or learning.

To help maintain participants’ attention during the task, a target detection task was incorporated into the procedure. Healthy participants were asked to press the space bar when they detected an abnormally high-pitched syllable, while patients were instructed to imagine holding their right hand when they detected the high-pitched syllable. The target tones were acoustically distinct from the embedded sequences and occurred infrequently. This manipulation was designed to draw the patients’ attention toward the auditory stream via a physically salient stimulus. No behavioral responses were required, and therefore whether the patients actually performed this detection remains unknown. Performance on this task did not depend on learning the statistical regularities, and participants remained unaware of the embedded structure.

### 2.4 EEG recording and preprocessing

EEG signals were recorded at a sampling rate of 1,000 Hz using a 32-electrode BrainCap in accordance with the standard 10–20 International System (Brain Products GmbH, Munich, Germany). One electrode was positioned beneath the right eye to record the electrooculogram. The EEG signals were initially referenced online to the FCz channel. All subsequent pre-processing steps were performed using MATLAB R2024b scripts and the EEGLAB toolbox (version 2024.1). Filtering was applied using a fifth-order Butterworth IIR filter with zero-phase forward-backward filtering (filtfilt, MATLAB). The filtering sequence consisted of a high-pass filter at 0.5 Hz, a notch filter at 48–52 Hz, followed by a low-pass filter at 40 Hz. Edge artefacts were minimized by the default reflection padding implemented in MATLAB’s filtfilt function. Independent Component Analysis was then performed using the ICLabel algorithm, and the components associated with ocular, muscular, and cardiac artefacts were identified and removed. The EEG data were re-referenced offline to the average mastoid recording (left and right mastoid), and the sampling rate was reduced to 80 Hz. Finally, the 10-min continuous data were divided into 50 segments, each equivalent to the duration of 16 trisyllabic words and lasting 12 s.

### 2.5 Phase coherence analyses

To focus on the steady-state responses, the random syllables presented at the beginning and end of each speech sequence were excluded from the analysis. The segmented single-trial EEG data were then transformed into the frequency domain using the discrete Fourier transform (DFT). The DFT was always performed on an individual epoch.

Neural entrainment was quantified by measuring inter-trial phase coherence (ITPC) at specific frequencies. This is defined as follows:

$$\text{ITPC}(f)=\frac{1}{n}(\sum_{k=1}^{n}\cos{\left(\theta_{k}\right)}^{2}+\sum_{k=1}^{n}\sin{\left(\theta_{k}\right)}^{2})$$

Values range from 0, indicating purely non-phase-locked activity, to 1, indicating strict phase-locked activity. Higher ITPC values reflect a more consistent phase alignment of neural responses at the specified frequency across multiple trials, thus indicating enhanced neural synchronization. We focused on three frequencies corresponding to linguistically meaningful units in the speech sequences: the word rate (i.e., 1.33 Hz), the frequency of the second harmonic of the word rate (i.e., 2.67 Hz), and the syllable rate (i.e., 4 Hz). The ITPC at each frequency was averaged across all electrodes.

In addition, we quantified participants’ word learning efficiency using the Word Learning Index (WLI) (29, 40), which was calculated as follows:

$$\mathrm{WLI}=\frac{{\text{ITPC}}_{\text{word frequency}}}{{\text{ITPC}}_{\text{syllable frequency}}}$$

This relative measure is more sensitive to subtle changes in the word learning process because it does not rely solely on the baseline amplitude of word or syllable frequency. Higher WLI values suggest successful learning of the artificial word, as they indicate greater neural entrainment at the word frequencies compared to the individual syllable frequencies.

### 2.6 Statistical analyses

To examine neural entrainment at the target frequencies (i.e., 1.33 Hz, 2.67 Hz, and 4 Hz), we first conducted a Wilcoxon matched-pairs signed-rank test to determine whether the ITPC at each target frequency was significantly stronger than the average of the four neighboring frequency bins. Specifically, the two bins directly below and the two bins directly above the target bin (with a bin width of 0.0833 Hz each) were averaged to obtain the power estimate. Bonferroni correction was applied to control for multiple comparisons.

To assess the participants’ SL ability more directly, we used the WLI to represent the word learning effect in the following analyses. Given the potential variability and fluctuations in consciousness among patients with DoC, the 10-min EEG recording was divided into five 2-min segments (i.e., 0.00–119.99, 120.00–239.99, 240.00–359.99, 360.00–479.99, and 480.00–599.99 s) to capture the temporal dynamics of neural activity. This generated sequential temporal segments for analysis. WLI values were then calculated for each 2-min window. The first 2-min window was designated as the baseline for each participant. The one with the maximum WLI value among the subsequent time windows was selected to represent each participant’s peak SL performance for subsequent comparison. A Wilcoxon matched-pairs signed-rank test was then performed on the baseline and the maximum WLI values, and the difference was used as an index of the SL effect.

To explore the clinical value of EEG-based SL metrics in patients with DoC, we conducted Spearman correlation analysis on the WLI-difference and the CRS-R scores obtained at the time of the EEG recording and at the 6-month follow-up. The WLI-difference is defined as the change in WLI from baseline to maximum value across the 2-min time windows.

Finally, we investigated whether narrow-band EEG synchronization related to SL across specific frequency bands could serve as a biomarker for the classification of patients with DoC. We conducted predictive modelling using a generalized linear model (GLM), which was implemented in the caret package (version 7.0.1) in R (version 4.5.2). Two models were constructed: the first classified HC and patients with DoC, and the second differentiated MCS from UWS. Both models used frequency-specific ITPC features as predictors, including baseline ITPC (i.e., the mean ITPC value in the first 2-min time window) and maximum ITPC (i.e., the highest ITPC value across the remaining time windows) at 1.33 Hz, 2.67 Hz, and 4 Hz. They were centered and scaled prior to model training. Model generalizability was assessed through ten-fold cross-validation. To evaluate the significance of the models, we performed permutation tests (1,000 iterations) by shuffling the dependent variables. To predict patient outcome, we included all patients from baseline data collection. Here, “positive” outcome was defined as an increase in CRS-R total score from baseline to follow-up, whereas “negative” outcome was defined as a decrease or no change in score during the same period. “unknown” outcome referred to patients for whom follow-up data could not be obtained due to reasons such as mortality.

## 3 Results

### 3.1 EEG neural tracking effects

**Figure 2:**
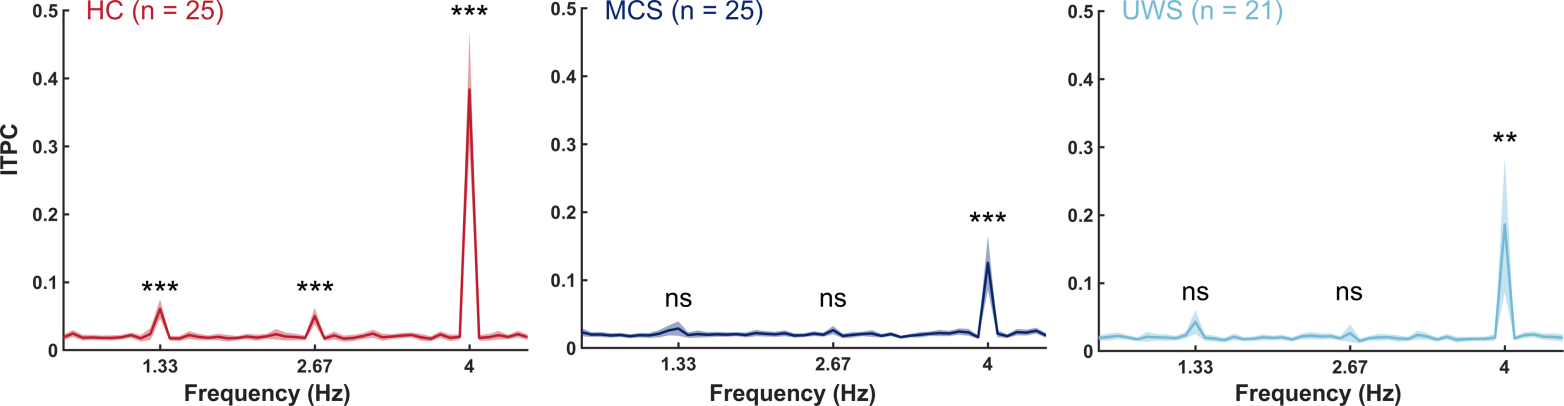
Group-averaged ITPC spectra at the syllable and word rates (Wilcoxon matched-pairs signed-rank test). ITPC spectral responses were averaged over the 10-min exposure period for the HC (*n* = 25), MCS (*n* = 25), and UWS (*n* = 21) groups. The shaded area covers 2 standard errors across participants. HC, healthy control; MCS, minimally conscious state; UWS, unresponsive wakefulness syndrome; ns, not significant. **p < 0.01, ***p < 0.001 (Bonferroni corrected *p* value threshold).

The EEG responses averaged over the 10-min exposure period are shown in Figure 2 and Table 2. ITPC spectral responses at the word and syllable rates were compared with their adjacent frequencies. For the HC group, significant response peaks were observed at the word rate (i.e., 1.33 Hz; *Z* = 4.184, Bonferroni-corrected *p* = 8.70 × 10^−5^), the rate corresponding to the second harmonic of the word (i.e., 2.67 Hz; *Z* = 3.754, Bonferroni-corrected *p* = 5.22 × 10^−4^) and the syllable rate (i.e., 4 Hz; *Z* = 4.372, Bonferroni-corrected *p* = 3.60 × 10^−5^). In the MCS and UWS groups, a significant response was also found at the syllable rate (MCS: *Z* = 4.238, Bonferroni-corrected *p* = 3.60 × 10^−5^; UWS: *Z* = 3.285, Bonferroni-corrected *p* = 3.00 × 10^−3^), but not at the word (*Z* < 1.236, Bonferroni-corrected *p* > 0.081) or its second harmonic rate (*Z* < 1.359, Bonferroni-corrected *p* > 0.522). We also conducted a region-of-interest (ROI) analysis using ten electrodes (Fz, F3, F4, FC1, FC2, Cz, C3, C4, CP1, and CP2) that are commonly implicated in language and statistical learning processing based on prior literature (29, 41). The detailed results are provided in Supplementary Table 1. The ROI results were qualitatively identical to the whole-brain average, indicating that the observed effects are robust and not driven by any specific electrode subset.

**Table 2:** ITPC neural tracking effects of the target frequencies across three groups (Wilcoxon matched-pairs signed-rank test).

Frequency	HC		MCS		UWS	
	*Z-*value	*P*-value	*Z*-value	*P*-value	*Z-*value	*P*-value
1.33 Hz	4.184	8.70 × 10^−5^***	0.821	1.000	2.207	0.081
2.67 Hz	3.754	5.22 × 10^−4^***	1.359	0.522	0.365	1.000
4 Hz	4.372	3.60 × 10^−5^***	4.238	3.60 × 10^−5^***	3.285	3.00 × 10^−3^**

HC, Healthy Control; MCS, minimally conscious state; UWS, unresponsive wakefulness syndrome; **P < 0.01, ***P < 0.001 (Bonferroni corrected P value threshold).

### 3.2 EEG statistical learning effects

**Figure 3:**
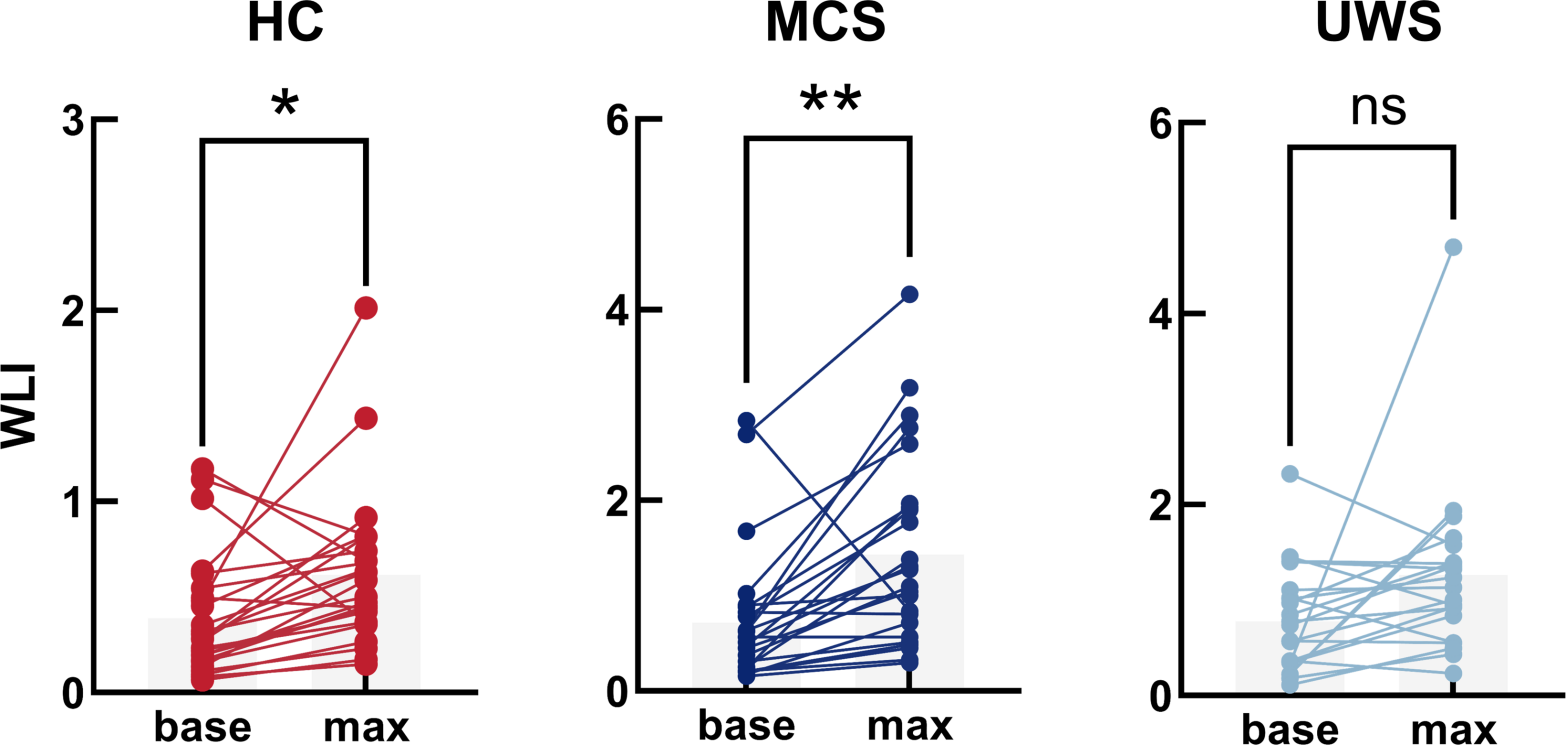
Comparison of baseline and maximum WLI values across groups (Wilcoxon matched-pairs signed-rank test). In each inset, the dot on the left represents the baseline WLI value from an individual participant, while the dot on the right represents the participant’s maximum WLI value. Lines between paired dots depict the WLI changes for an individual participant across time. The grey bars indicate group-averaged WLI values. WLI, Word Learning Index; HC, healthy control; MCS, minimally conscious state; UWS, unresponsive wakefulness syndrome; ns = not significant; base, the baseline WLI value; max, the maximum WLI value. *p < 0.05, **p < 0.01 (Bonferroni corrected *p* value threshold).

Following the ITPC spectral analysis, we further computed the WLI, defined as the ratio of ITPC at 1.33 Hz to that at 4 Hz. This ratio normalizes individual differences in overall brain activity between participants, providing a more comparable measure. To account for possible fluctuations in consciousness, we divided the 10-min recording into five 2-min segments for analysis. The results of the Wilcoxon signed-rank tests performed on the baseline and the maximum WLI values for each group are displayed in Figure 3. Significant differences between the baseline and the maximum WLI values were observed both in the HC group (*Z* = 2.758, Bonferroni-corrected *p* = 0.018) and in the MCS group (*Z* = 3.700, Bonferroni-corrected *p* = 0.000), but not in the UWS group (*Z* = 2.103, Bonferroni-corrected *p* = 0.105). This finding suggests that both the MCS group and the HC group exhibited significant responses to statistical regularities. Furthermore, given that patients with MCS are defined by the presence of residual minimal consciousness, the results suggest that the SL process may require a minimal level of conscious awareness.

### 3.3 Correlation between statistical learning effect and behavioral scores of patients with DoC

**Figure 4:**
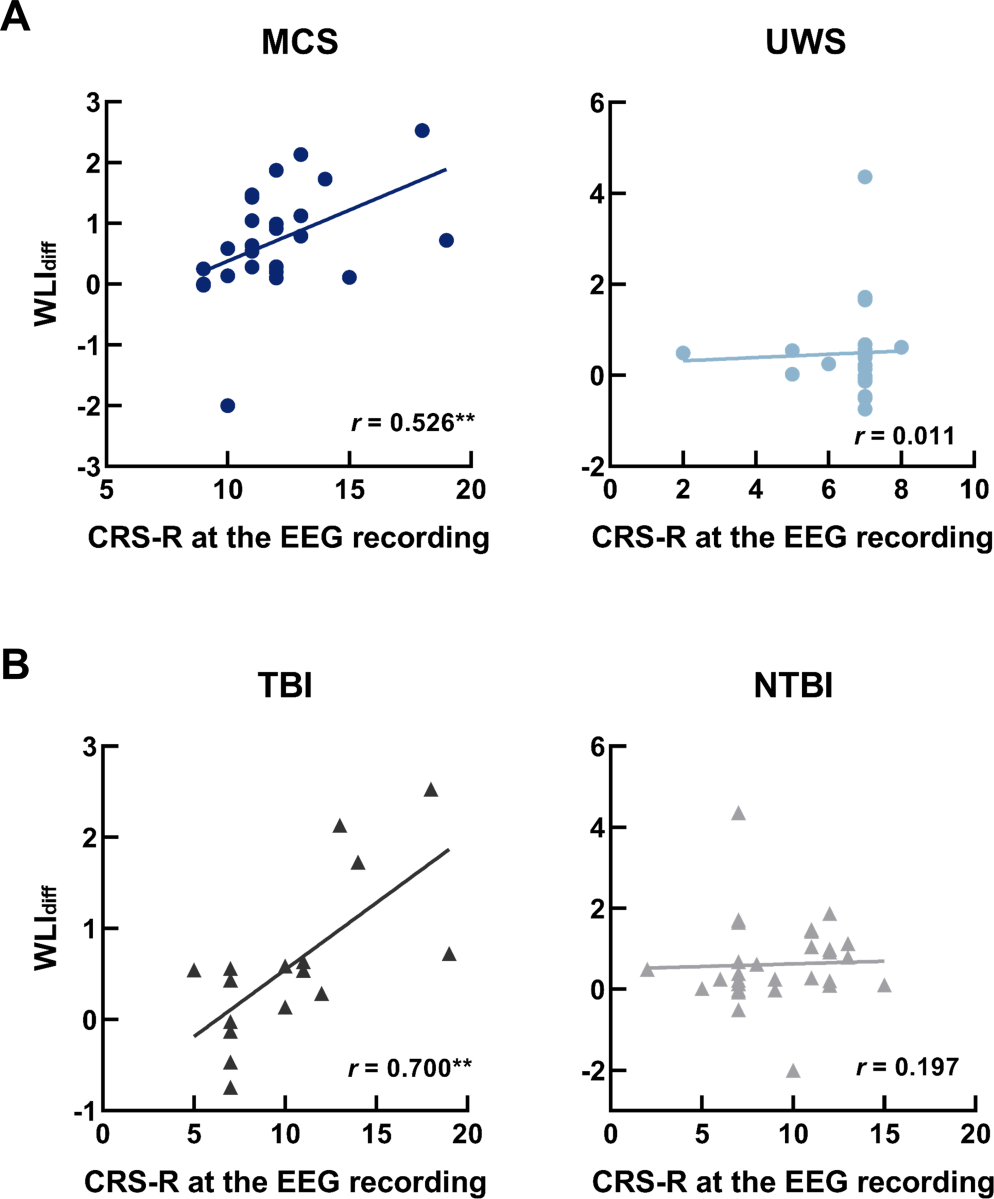
Spearman correlation analyses between the WLI-difference and the CRS-R scores at the time of EEG recording. In each inset, a filled circle represents an individual patient measurement. **(A)** A significant positive correlation between WLI_diff_ and CRS-R scores at the time of EEG recording was observed in the MCS subgroup but was absent in the UWS subgroup. **(B)** A significant correlation between WLI_diff_ and CRS-R scores at the time of EEG recording was observed in the TBI subgroup but was absent in the NTBI subgroup. WLI_diff,_ the change in Word Learning Index from the baseline to the maximum value across the 2-min time windows; CRS-R, Coma Recovery Scale-Revised; DoC, disorders of consciousness; MCS, minimally conscious state; UWS, unresponsive wakefulness syndrome. **p < 0.01.

We first calculated the WLI-difference, which is defined as the change in WLI from baseline to its maximum value over time windows. We then conducted Spearman correlation analyses between WLI-difference and CRS-R scores of patients with DoC at the time of EEG recording. The results are shown in Figure 4. A significant correlation between WLI_diff_ and CRS-R scores at the time of EEG recording was found in the MCS group (*r(23)* = 0.526, *p* = 0.007) (Figure 4A). This suggests that patients with MCS who demonstrated stronger neural tracking of statistical regularities were more likely to exhibit better behavioral results. In the UWS group, the correlation was not significant (*r(19)* = 0.011, *p* = 0.961). We also analyzed the correlation between WLI_diff_ and CRS-R scores at the time of EEG recording for patients across etiology (Figure 4B). A significant positive correlation was observed in the TBI subgroup (*r(14)* = 0.700, *p* = 0.003). The correlation was not significant in the NTBI subgroup (*r(28)* = 0.197, *p* = 0.298).

**Figure 5:**
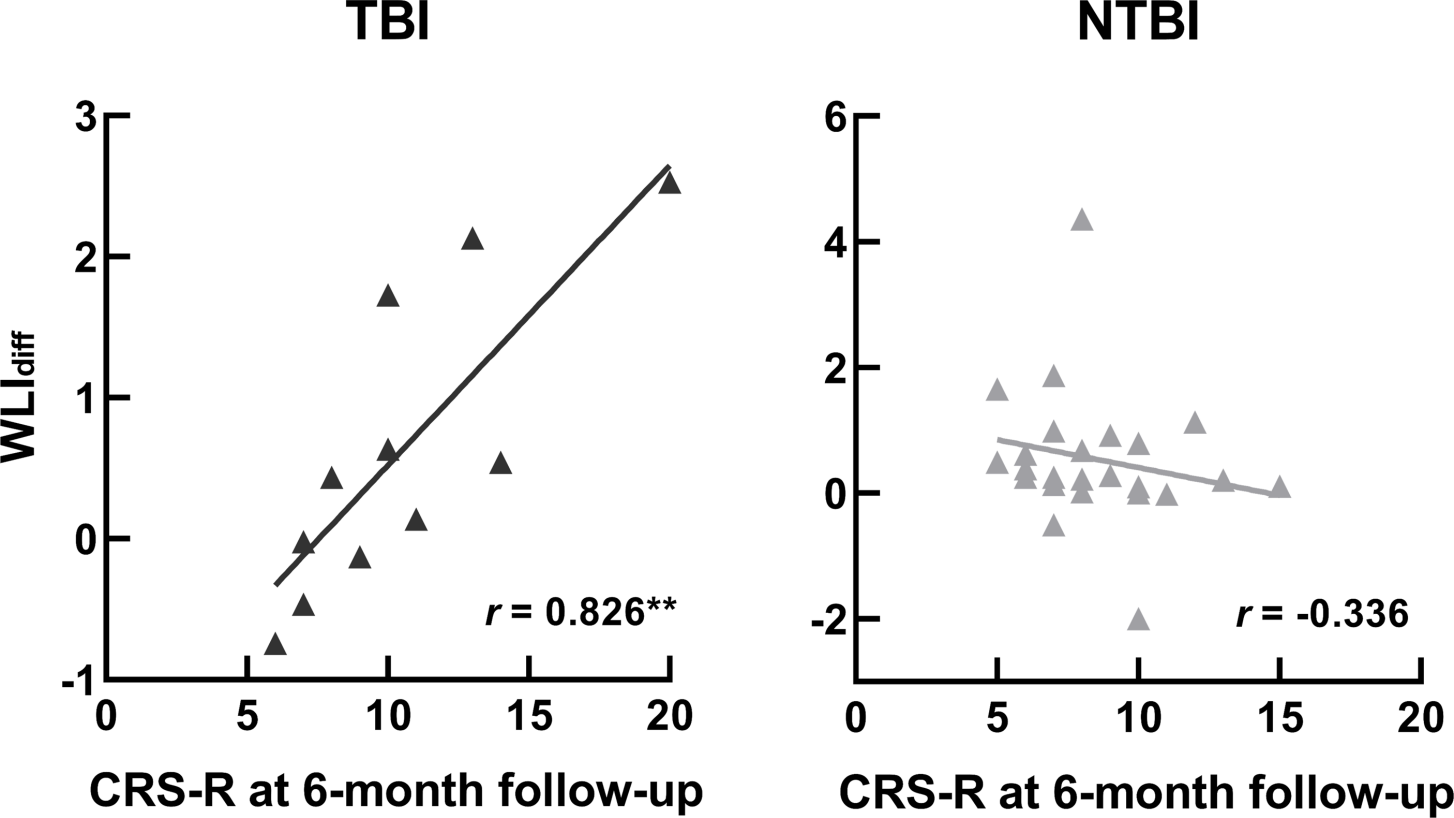
Spearman correlation analyses between the WLI-difference and the CRS-R scores at 6-month follow-up. In each inset, a filled circle represents an individual patient measurement. A significant correlation between the WLI_diff_ and CRS-R scores at the 6-month follow-up was observed in the TBI subgroup. However, no such relationship was found in the NTBI subgroup. WLI_diff_, the change in Word Learning Index from the baseline to the maximum value across the 2-min time windows; CRS-R, Coma Recovery Scale-Revised; DoC, disorders of consciousness; TBI, traumatic brain injury; NTBI, non-traumatic brain injury. **p < 0.01.

When analyzed against the CRS-R scores at 6 months, WLI-difference showed a significant association with CRS-R scores at 6-month follow-up in the TBI group (*r(14)* = 0.826, *p* = 0.002). No such correlations were observed in the NTBI, MCS, or UWS groups (*r* < 0.004, *p* > 0.109) (Figure 5; Table 3). These findings highlight the influence of etiology in relation to the prognosis.

**Table 3:** Spearman correlation analyses between changes in WLI values and CRS-R scores for patients with DoC.

Learning effects	Groups	CRS-R scores at the EEG recording		CRS-R scores at 6-month follow-up	
		*r*-value	*P*-value	*r*-value	*P*-value
WLI_diff_	MCS	0.526	0.007**	0.004	0.986
	UWS	0.011	0.961	−0.099	0.716
	TBI	0.700	0.003**	0.826	0.002**
	NTBI	0.197	0.298	−0.336	0.109

CRS-R, Coma Recovery Scale-Revised; DoC, disorders of consciousness; WLI_diff_, the change in Word Learning Index from the baseline to the maximum value across the 2-min time windows; MCS, minimally conscious state; UWS, unresponsive wakefulness syndrome; TBI, traumatic brain injury; NTBI, non-traumatic brain injury; **p < 0.01.

### 3.4 The clinical application of the statistical learning effects

**Figure 6:**
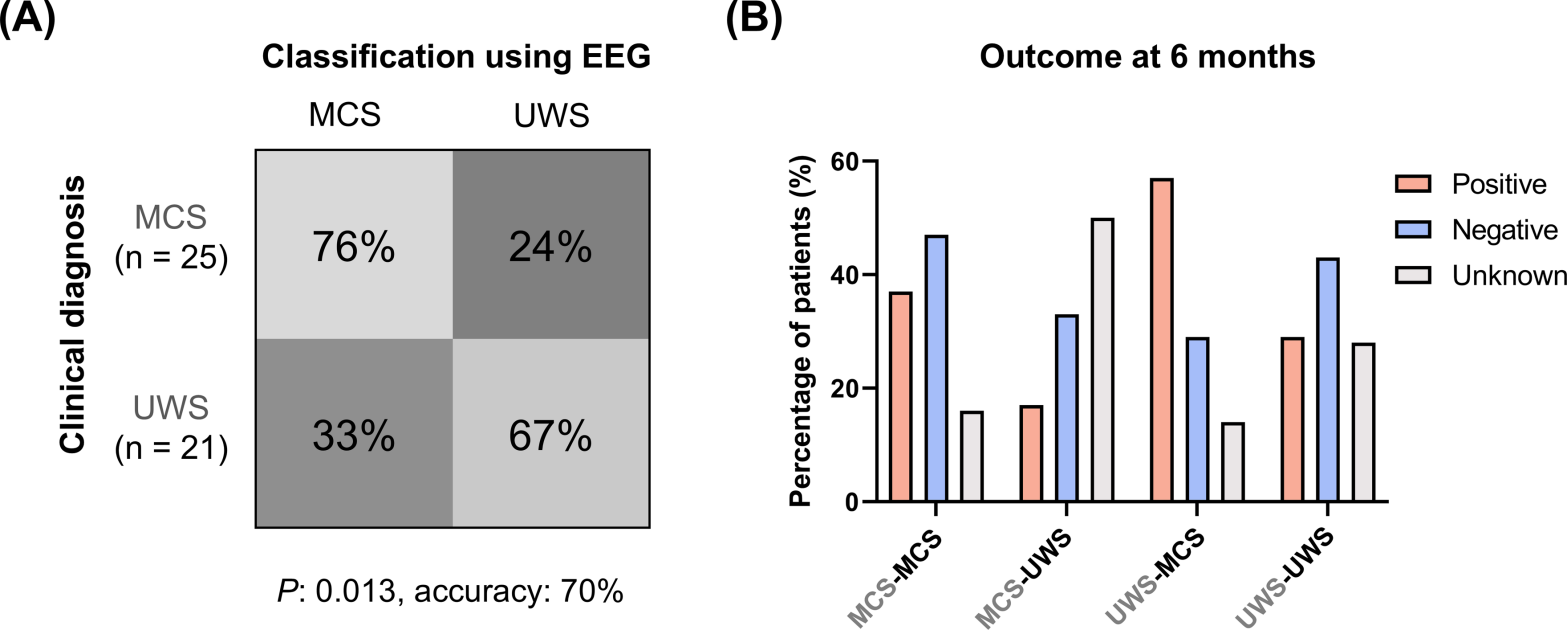
**(A)** Confusion matrix of consciousness diagnosis generated by the LDA with statistical significance assessed by permutation test (1,000 iterations). Among patients clinically assessed as MCS, 76% (19/25) were classified as MCS by the EEG model, while 24% (6/25) were classified as UWS. Among patients clinically diagnosed as UWS, 33% (7/21) were identified as MCS by the EEG model, while 67% (14/21) were classified as UWS. **(B)** The proportions of 6-month outcomes in the UWS and MCS groups defined by the confusion matrix in panel A. Light grey labels denote the baseline behavioral diagnosis, while black labels denote the baseline EEG diagnosis. The percentages in each group show the 6-month outcomes (positive, negative, or unknown) for patients in the respective diagnostic subgroup. MCS, minimally conscious state; UWS, unresponsive wakefulness syndrome.

To assess the clinical potential of statistical learning effects, we trained two GLM classifiers with ten-fold cross-validation. Both classifiers demonstrated significant effectiveness. The models accurately classified the HC group and patients with DoC (ACC = 76%, *p* < 0.001) and distinguished MCS from UWS (ACC = 70%, *p* = 0.013). Figure 6A presents the confusion matrix for the MCS and UWS classification. The decoder correctly identified MCS and UWS patients with accuracies of 76 and 67%, respectively, both exceeding the chance level of 50%.

Of note, a proportion (33%, 7/21) of patients who were clinically diagnosed with UWS based on CRS-R were classified as MCS using the EEG-based model, which may reflect residual consciousness not identified by the behavioral CRS-R evaluation. Interestingly, as shown in Figure 6B, the rate of behavioral improvements at the 6-month follow-up was higher in such potentially misdiagnosed patients with UWS (57%, 4/7) than in those diagnosed as UWS by both the CRS-R and EEG classifier methods (28%, 4/14). Conversely, a proportion (24%, 6/25) of patients labelled as MCS by the CRS-R were classified as UWS by the EEG model. These potentially misdiagnosed MCS patients showed a lower rate of behavioral improvement after 6 months (17%, 1/6), compared to those with a consistent MCS diagnosis by both methods (37%, 7/19). These observations suggest that integrating information from both behavioral assessments and SL-related EEG classifications may provide a more informative perspective on potential outcomes.

## 4 Discussion

This study used a passive auditory paradigm involving trisyllabic pseudowords to investigate SL abilities in patients with DoC. Combining the EEG technique with frequency-tagging analysis enabled us to capture neural responses to underlying statistical structures with precision. A significant within-group effect for statistical regularities in auditory input (e.g., TP between syllables) was found in the HC group and in the MCS group. In the UWS group, the effect was not significant. These findings showed that SL may require only a minimal level of conscious involvement. In addition, our results demonstrated that the EEG-based SL effect was significantly correlated with the CRS-R scores at the time of EEG recording in the MCS group, whereas this correlation was not significant in the UWS group. We further examined the effect of etiology and found that, in patients with TBI, the SL effect was significantly associated with CRS-R scores at the time of EEG recording and at the 6-month follow-up, while the associations were not significant in patients with NTBI. Finally, the corresponding EEG models highlight the EEG signals reflecting SL ability as indicators of residual consciousness in patients with DoC.

### 4.1 MCS patients retain the ability of statistical learning

The WLI measure captured learning-related neural dynamics, with significant changes observed in healthy controls. In the MCS group, significant WLI changes were also found, reflecting neural responses to SL. In the UWS group, no significant WLI changes were found. This peak-selected analysis may be subject to winner’s curse bias. Nevertheless, given the large individual differences in the timing of learning effects and the fluctuating conscious state of patients with DoC, we considered this approach to be a reasonable compromise for capturing each participant’s best response. This finding is consistent with previous research (31, 32). SL involves extracting distributional regularities from environmental input with particular sensitivity to TP between elements (42). For example, when exposed to continuous syllable streams (e.g., “pa-do-ti-bi-da-ku-go-la-bu-bi-da-ku…”), healthy individuals can reliably detect that “da” consistently follows “bi,” whereas “ku” can be followed by different syllables (e.g., “go” or “pa”) in a syllable stream (23, 29). Despite their reduced and fluctuating levels of consciousness, patients with MCS retain functional coordination in the main brain regions involved in residual language processing, such as the superior temporal gyrus, the left middle temporal gyrus, and the left angular gyrus (30, 43, 44). Thus, they retain the ability to track the regularities of adjacent elements, and their residual consciousness supports the integration of these transitional cues into larger units. In contrast, patients with UWS, who typically have more severe brain injuries, exhibit disruption to network coordination dependent on conscious processing (30, 45). Even if early auditory responses (e.g., N100 potentials) are preserved, signal transmission to higher-order associative cortices is often impaired due to disrupted connectivity (46, 47). Consequently, patients with UWS are unable to engage in top-down attentional modulation or support higher-level pattern integration. However, in the ITPC analysis, the MCS group did not show a significant response at the word rate. Notably, the MCS group was predominantly composed of MCS- patients (i.e., MCS without evidence of language function; *n* = 21) with only a small subset of MCS + patients (i.e., MCS with evidence of language function; *n* = 4) in the present study. This unbalanced composition likely contributed to the null finding, as MCS- patients lack high-level behavioral responses such as command-following and intentional communication and may therefore exhibit particularly impaired neural tracking of linguistic structures (48). Future studies with more balanced MCS- and MCS + samples are needed. In addition, ITPC can be modulated by signal power and signal-to-noise ratio, and these factors were not strictly controlled for in the present study.

### 4.2 Complex statistical learning requires minimal consciousness

This study provides new evidence regarding the relationship between consciousness and SL. Patients with DoC are characterized by marked heterogeneity in residual consciousness. Patients with MCS are generally considered to retain fluctuating consciousness and some preserved cognitive processing, whereas patients with UWS lack signs of consciousness (4, 5, 49, 50). In the present study, we found that patients with MCS retain SL capacity, as demonstrated by their ability to progressively show neural entrainment to structured pseudoword sequences. Patients with UWS did not show significant neural entrainment. This dissociation highlights the need for minimal conscious network integration for effective SL. Moreover, within the MCS group, greater behavioral scores were associated with stronger learning responses, indicating that subtle differences in residual consciousness and cognitive resources can influence the neural efficiency of SL. Taken together, these findings suggest the possibility of a two-stage account of SL, whereby consciousness contributes differently at distinct processing stages. In stage 1 (unconscious detection), primary sensory regions (e.g., the auditory cortex) automatically identify local statistical regularities, such as the probability of syllable transitions. This process appears to be preserved in patients with UWS, as measured by ITPC at the syllable rate (i.e., 4 Hz) observed in this study. Therefore, fully automatic SL processing may primarily capture stage 1 without engaging higher-order integrative mechanisms. In contrast, stage 2 depends on the conscious integration. This stage requires minimal conscious engagement, as demonstrated by the progressive increase in WLI values observed in patients with MCS, which reflects the consolidation of learned patterns. The residual consciousness of patients with MCS may enable them to intermittently reactivate integration networks, allowing for partial WLI progression despite fluctuating attention. This highlights that the demand for consciousness increases with SL task complexity.

### 4.3 Statistical learning EEG metrics have diagnostic and prognostic potential for patients with DoC

The present paradigm offers several practical advantages for the potential of clinical application. It is capable of detecting awareness that may be obscured by motor or behavioral disorders, bedside applicable, and requires only a brief 10-min recording session. These features are particularly valuable in patients with DoC, whose motor impairment and fluctuating arousal often preclude standard neuropsychological testing. Our previous work established the clinical utility of explicit learning paradigms for diagnosis and prognosis (37). The current study approaches this issue from a different angle by examining implicit learning, which operates without deliberate engagement. We demonstrate that this implicit mechanism also yields neural indices capable of distinguishing conscious states, thereby broadening the scope of learning-based neurophysiological markers for clinical assessment.

The present findings specifically highlight the potential of this paradigm as an adjunct screening tool, providing insights into the clinical application that had not been addressed in prior studies (31, 32). In this study, we found that, within the MCS group, individuals with stronger learning effects tended to demonstrate greater behavioral scores at the time of EEG recording. No significant association was observed among patients with UWS. Furthermore, additional analysis of the CRS-R subscales revealed that, in the MCS group, EEG indices were significantly correlated with both the language and motor subscales (see in Supplementary Table 2), whereas no such correlations were observed in the UWS group. Although this suggests relatively preserved language and sensorimotor processing in patients with MCS, the lack of structural neuroimaging data (e.g., MRI) precludes determining whether this association reflects focal preservation of task-relevant regions or more diffuse network integrity. It is worth noting that the absence of significant correlations in the UWS group may partly reflect minimal inter-individual variability in CRS-R scores (51, 52). Consequently, this finding should not be interpreted as evidence that the EEG-based neural tracking measure is completely unrelated to CRS-R scores in UWS patients. Building on the above findings, we developed predictive models using ITPC features to assess their potential clinical value in classification. The results showed that the models could distinguish HC from patients with DoC, as well as differentiate patients with MCS from UWS, providing neurophysiological support for the view that patients with MCS retain partially preserved high-order information processing capacities (30). Importantly, a subset of patients behaviorally diagnosed as UWS were classified as MCS by the EEG model, raising the possibility of covertly conscious states. We further characterized the misclassified patients and found no significant correlations between the WLI_diff_ values and clinical variables (including CRS-R total and subscale scores, age and time since injury) within either misclassification subgroup. This lack of commonality suggests that the discrepancy between EEG-based classification and behavioral diagnosis does not arise from any single, easily identifiable clinical factor. Rather, the neural entrainment is jointly shaped by multiple factors, such as residual cognition, lesion distribution, compensatory mechanisms, and other variables (30, 53). Future multimodal investigations combining EEG with functional magnetic resonance imaging may help to determine whether these patients meet criteria for cognitive motor dissociation (9).

We also demonstrated the clinical utility of SL-related EEG signals in exploring the potential relevance to prognosis in patients with DoC. The results suggested that combining SL-related EEG metrics with the traditional behavioral assessments could provide a more reliable framework for assessing 6-month outcomes. When behavioral assessments such as the CRS-R are ambiguous or yield controversial results, this paradigm may serve as an objective, supplementary source of neurophysiological evidence to support diagnostic and prognostic decisions. It may also provide a temporal window for rehabilitation intervention, as the demonstration of SL capacity may indicate greater recovery potential. Moreover, the observed associations between SL effects and behavioral scores were strongly dependent on etiology. Specifically, we observed a significant positive correlation between the SL effects and CRS-R scores at both the time of EEG recording and the 6-month follow-up in the TBI subgroup. In the NTBI subgroup, the correlation did not reach statistical significance. This difference can be explained by the distinct patterns of neural damage associated with each injury type. In the TBI group, diffuse axonal injury typically leaves the overall structure of major brain networks largely intact (7). By contrast, NTBI, which is often caused by oxygen deprivation or metabolic dysfunction, typically involves more widespread cellular damage and microvascular changes (54). These findings align with previous studies showing that traumatic causes of DoC are linked to more favorable outcomes, indicating that etiology is a significant predictor of outcome (55–57). Therefore, future studies may benefit from prioritizing etiology as a key factor in diagnostic and prognostic assessments.

### 4.4 Limitations

This study has several limitations that should be taken into account. First, the relatively small sample size and the lack of external validation may reduce the statistical power to detect subtle differences in statistical learning abilities, which requires validation in larger longitudinal cohorts before any clinical implementation can be considered. Second, a limitation of our approach is the lack of sliding windowing and random control, which restricts our ability to track rapid changes in neural entrainment over time and to isolate SL effects from low-level acoustic confounds. In addition, the target detection task does not fully dissociate attentional engagement from SL, so we cannot exclude attentional contributions to the observed effects. Future studies could employ refined paradigms to directly dissociate these contributions. Another limitation is that the stimuli contained acoustic properties that may introduce confounding factors, potentially obscuring the specific contribution of statistical structure. Finally, the paradigm focused solely on the auditory modality and EEG technique, making it unclear whether effects reflect focal or diffuse network integrity. Future studies could integrate multimodal stimuli (e.g., tactile or visual SL tasks) and neuroimaging to improve generalizability and clarify the underlying neural basis.

## 5 Conclusion

In conclusion, we present evidence of SL in the MCS group using a passive auditory paradigm. The within-group effect was significant in both the HC group and the MCS group, but not in the UWS group. Furthermore, when combined with behavioral assessments, these EEG markers could provide a more reliable framework for assessing 6-month outcomes. These findings indicate that SL may be associated with residual consciousness in the integration of structured regularities and highlight the potential of SL-related EEG measures as an objective neural index for clinical assessment and prognosis in patients with DoC.

## Data Availability

The raw data supporting the conclusions of this article will be made available by the authors, without undue reservation.

## Footnotes

1
http://www.neospeech.com/
