# Supplementary material for: The diagnostic and prognostic value of auditory statistical learning ability in patients with disorders of consciousness
Source: Front Neurol. 2026 Jul 22;17:1841501. doi: 10.3389/fneur.2026.1841501 (PMC13437382; doi:10.3389/fneur.2026.1841501)
Supplement: Supplementary file 1 [file Supplementary_file_1.docx]

*Supplementary Material*

**Supplementary Tables**

**Supplementary Table 1 ITPC neural tracking effects of the target frequencies across three groups at regions of interest (Wilcoxon matched-pairs signed-rank test).**

| **Frequency** | **HC** | | **MCS** | | **UWS** | |
| --- | --- | --- | --- | --- | --- | --- |
|  | ***Z-*value** | ***P*-value** | ***Z*-value** | ***P*-value** | ***Z-*value** | ***P*-value** |
| **1.33 Hz** | 3.861 | 3.39 × 10^-4^*** | 0.686 | 1.000 | 2.242 | 0.075 |
| **2.67 Hz** | 3.565 | 0.001** | 1.413 | 0.473 | 1.060 | 0.867 |
| **4 Hz** | 4.345 | 4.20 × 10^-5^*** | 4.023 | 1.74 × 10^-4^*** | 3.389 | 0.002** |

HC = Healthy Control; MCS = minimally conscious state; UWS = unresponsive wakefulness syndrome; ***P* < 0.01, ****P* < 0.001(Bonferroni corrected *P* value threshold).

**Supplementary Table 2 Spearman correlation analyses between changes in WLI values and CRS-R subscale scores for patients with DoC.**

| **Learning effects** | **Group** | | **CRS-R Tot** | **CRS-R AF** | **CRS-R VF** | **CRS-R MF** | **CRS-R OF** | **CRS-R C** | **CRS-R Ar** |
| --- | --- | --- | --- | --- | --- | --- | --- | --- | --- |
| **WLI_diff_** | **MCS** | *r*-value | 0.526 | 0.094 | 0.067 | 0.402 | 0.411 | 0.028 | 0.278 |
|  |  | *P*-value | 0.007** | 0.654 | 0.749 | 0.046* | 0.041* | 0.893 | 0.179 |
|  | **UWS** | *r*-value | 0.011 | -0.113 | -0.295 | -0.111 | 0.271 | - | -0.083 |
|  |  | *P*-value | 0.961 | 0.625 | 0.195 | 0.633 | 0.235 | - | 0.179 |

CRS-R = Coma Recovery Scale-Revised; DoC = disorders of consciousness; WLI_diff_ = the change in Word Learning Index; MCS = minimally conscious state; UWS = unresponsive wakefulness syndrome; CRS-R Tot = Total score of the CRS-R scale; CRS-R AF = Auditory Function subscale of the CRS-R scale; CRS-R VF = Visual Function subscale of the CRS-R scale; CRS-R MF = Motor Function subscale of the CRS-R scale; CRS-R OF = OroMotor/Verbal Function subscale of the CRS-R scale; CRS-R C = Communication subscale of the CRS-R scale; CRS-R Ar = Arousal subscale of the CRS-R scale; **P* < 0.05, ***P* < 0.01.
